# Beyond Adaptive Mental Functioning With Pain as the Absence of Psychopathology: Prevalence and Correlates of Flourishing in Two Chronic Pain Samples

**DOI:** 10.3389/fpsyg.2019.02443

**Published:** 2019-11-05

**Authors:** Hester R. Trompetter, Floortje Mols, Gerben J. Westerhof

**Affiliations:** ^1^CoRPS – Center of Research on Psychological and Somatic disorders, Department of Medical and Clinical Psychology, Tilburg University, Tilburg, Netherlands; ^2^Centre for eHealth and Wellbeing Research, Department of Psychology, Health and Technology, University of Twente, Enschede, Netherlands

**Keywords:** chronic pain, positive mental health, flourishing, depression, psychological flexibility, values, fear avoidance, resilience

## Abstract

Chronic pain outcomes are traditionally defined in terms of *dis*ability and *illness*. A definition of adaptive functioning in the context of chronic pain beyond the mere absence of negative outcomes, is the ability to *flourish* (i.e., experience emotional, psychological and social *well-being*; Keyes, 2002). We explored in two chronic pain samples the prevalence and sociodemographic, physical and psychological correlates of flourishing, and complemented this exploration with a similar examination of *(being at risk for) psychopathology* to help contextualize findings. Sample 1 (*n* = 1498) was a nationally representative sample. Subgroups included people with regular joint pain (1), regular joint pain and rheumatoid arthritis (2) and without chronic pain (3). Using chi-square tests we calculated the prevalence of both mental health outcomes and examined if people with or without chronic pain were more/less likely to flourish/at risk for psychopathology. Sample 2 (*n* = 238) concerned baseline data of a Randomized Controlled Trial on the effectiveness of Acceptance and Commitment Therapy for chronic pain (Trompetter et al., 2015b). We performed logistic regression analysis to identify flourishers/those at risk for depression. The Mental Health Continuum-Short Form was used to measure flourishing. The prevalence of flourishing was 34% (recurrent joint pain) and 38% (recurrent joint pain and arthritis) in sample 1, and 23% in sample 2. Compared to those without chronic pain, people with chronic pain were as likely to flourish, but more likely to be at risk for psychopathology. In sample 2, both flourishing and being at risk for depression were related foremost to *psychological* correlates. While engaged living was the most important correlate of flourishing, pain catastrophizing and psychological inflexibility were most important correlates of being at risk for depression. In conclusion, people with chronic pain *are able* to flourish. Findings suggest that positive and negative chronic pain outcomes function on two different continua, with potentially unique protective and risk factors. The Psychological Flexibility model provides pathways to explain both poor and optimal functioning in the presence of chronic pain. A better understanding of people with chronic pain who are able to flourish can be a fruitful endeavor to improve chronic pain models and interventions.

## Introduction

Traditionally we study the effects of chronic pain on health and well-being from a medical, disease-oriented approach. Chronic pain outcomes are defined in terms of *dis*ability, *poor* functioning, and *illness.* It is well-established that chronic pain is associated with a variety of such negative outcomes like depression (Breivik et al., 2006; Miller and Cano, 2009; de Heer et al., 2018). Healthy, adaptive functioning with persistent pain is considered to be achieved when these negative outcomes are absent. Subsequently, we primarily study and target involved *risk* processes like pain-related fear and pain catastrophizing (Sullivan et al., 1995; Gatchel et al., 2007; Jensen and Turk, 2014). In contrast, relatively few studies examined the unique involvement of *protective* processes like optimism, positive affect, purpose in life and social support in adaptive functioning with pain (but see, e.g., Karoly and Ruehlman, 2006; Smith and Zautra, 2008; Verduin et al., 2008; Finan et al., 2013; Boselie et al., 2014). Scholars proposed that more focus on protective factors is necessary. Additionally, they suggested to develop alternative conceptualizations of chronic pain *outcomes* that define adaptive functioning as more than the mere absence of negative outcomes (Sturgeon and Zautra, 2010; Goubert and Trompetter, 2017). Based on the work of Keyes (2002) and Goubert and Trompetter (2017) proposed the study of *positive mental health* in the context of pain. Optimal levels of positive mental health entail the experience of high levels of emotional well-being (presence of positive feelings like happiness), psychological well-being (optimal functioning in life through, e.g., purpose in life and positive social relationships) and social well-being (optimal social/community functioning through, e.g., social integration). Keyes conceptualizes those functioning with optimal levels of emotional as well as psychological and social well-being as *flourishing*. He considers *complete mental health* to be achieved when someone is not suffering from mental illness and is simultaneously flourishing. To measure the construct of flourishing, Keyes developed and validated the Mental Health Continuum-Short Form (MHC-SF) (Keyes et al., 2008; Lamers et al., 2011). First studies suggest that negative (pain disability, depression) and positive (emotional, psychological well-being) chronic pain outcomes are only moderately related to each other (Mangelli et al., 2002; Schleicher et al., 2005; Huber et al., 2008). This mirrors the outcomes of a larger body of studies in general and psychiatric populations, showing that mental illness and positive mental health measured with the MHC-SF or similar instruments are associated, but *different* constructs (Huppert and Whittington, 2003; Keyes, 2005b; Westerhof and Keyes, 2009; Weich et al., 2011; Trompetter et al., 2017). This implies that positive and negative chronic pain outcomes and their underlying risk and protective factors are not two sides of the same coin (Goubert and Trompetter, 2017). Understanding if there is a subgroup of chronic pain sufferers that is able to flourish, and unraveling the factors that differentiate the flourishers from those who do not flourish might proof a fruitful endeavor. It may broaden our current theoretical models that explain chronic pain adaptation primarily in terms of unsuccessful adaptation and risk. Hereby we can expose new routes for (psychological) treatment to support successful chronic pain adaptation. In this study we examine the prevalence and correlates of flourishing in people with chronic pain. What knowledge currently exists? Two large, representative national studies using the MHC-SF report flourishing prevalence rates of 18 and 37% respectively in the general adult population in the United States (Keyes, 2005b) and Netherlands (Schotanus-Dijkstra et al., 2016). Only two studies directly addressed *flourishing* in pain samples, and compared this to the likelihood to flourish in non-pain samples. Using data from the Canadian Community Health Survey, Gilmour (2015) identified that the people who ‘usually experienced severe levels of pain/discomfort,’ were significantly less likely to flourish compared to those with mild levels of pain/discomfort. Similarly, Keyes (2005a) showed that suffering from chronic physical conditions – including specific subgroups with chronic back pain and/or arthritis – was significantly and negatively associated with flourishing. First studies that assessed aspects of positive mental health like happiness or purpose in life also indicate that people with chronic pain experience lower levels of well-being than people without chronic pain (Schleicher et al., 2005; Finucane et al., 2012). To our best knowledge, no studies exist that examine the correlates of flourishing in people with chronic pain. We perform *post hoc* analyses on two existing datasets, each including different chronic pain samples. A first aim is to explore in a Dutch nationally representative sample the prevalence of flourishing in people with chronic pain, in comparison to people without chronic pain (Study 1). A second aim is to explore in another sample – that consists of people with chronic pain with different underlying etiologies seeking psychological help – the prevalence of flourishing, and identify sociodemographic, physical and psychological correlates of flourishing in the context of chronic pain (Study 2). In integration these two studies build on existing prevalence studies (Keyes, 2005a; Gilmour, 2015) by assessing different chronic pain samples and/or different cultural contexts. The second dataset provides us with the opportunity to assess key correlates from two leading theoretical models explaining chronic pain disability – the Fear Avoidance model of chronic pain (FA model: Crombez et al., 2012) and the Psychological Flexibility model of chronic pain (PF model: McCracken and Vowles, 2014) – in relation to flourishing. In *both* studies we complement the examination of the prevalence and correlates of flourishing with an examination of the prevalence and correlates of (*being at risk for) psychopathology.* This will help readers to contextualize the findings on flourishing as a positive chronic pain outcome, with psychopathology as a more familiar negative chronic pain outcome. Additionally, it provides an examination of the postulation that psychopathology and flourishing and their underlying correlates are related, but different. Our first hypothesis is that chronic pain is associated with a lower likelihood to flourish and higher likelihood to be at risk for psychopathology compared to people without chronic pain (Study 1: Keyes, 2005a; Miller and Cano, 2009; Gilmour, 2015; de Heer et al., 2018). We do expect, however, that a proportion of people with chronic pain *will* flourish in both studies. In Study 2, we examine the correlates of flourishing in the context of chronic pain. Overall, *psychological* correlates are expected to show stronger associations with the included mental health outcomes than *sociodemographic* and/or *physical* correlates like pain intensity and pain disability (Keyes, 2005b; Schleicher et al., 2005; Vowles et al., 2007; Huber et al., 2008; McCracken and Gutiérrez-Martínez, 2011; Schotanus-Dijkstra et al., 2016). More specifically, *psychological* correlates that will be assessed include pain catastrophizing, psychological inflexibility, mindfulness and engaged living. Pain catastrophizing is a key construct in the FA model of chronic pain (Crombez et al., 2012). Psychological inflexibility, mindfulness and engaged living are key constructs in the PF model of chronic pain (McCracken and Vowles, 2014). These models underlie Cognitive Behavioral Therapy and Acceptance and Commitment Therapy for chronic pain, respectively (Ehde et al., 2014; Veehof et al., 2016). The FA model has been developed theoretically in line with a traditional medical view of chronic pain *dis*ability. As, furthermore (aspects of) catastrophizing thinking styles are an important risk factor for depression in- and outside the context of chronic pain (Vowles et al., 2007; Aldao et al., 2010; Crombez et al., 2012), we expect that pain catastrophizing is associated primarily to being at risk for psychopathology. In contrast, the PF model is most aligned with positive mental health of both models, through its central focus on long-term engagement with personally valued activities of the person with persistent pain (Fledderus et al., 2012; McCracken and Vowles, 2014). We therefore particularly expect that engaged living is an important correlate of flourishing in people with chronic pain. Finally, psychological inflexibility is expected to be associated with both mental health outcomes but most strongly to being at risk for psychopathology. Although it is a central process within the more positive PF model, the construct as operationalized in this study – experiential avoidance of pain and cognitive fusion with pain-related thoughts and feelings – particularly relate(s) to pain catastrophizing and negative pain-related outcomes (Aldao et al., 2010; Wicksell et al., 2010b; McCracken and Vowles, 2014; Trompetter et al., 2015a).

## Study 1

### Materials and Methods

#### Participants and Procedure

In this paper, we make use of data of the LISS (Longitudinal Internet Studies for the Social sciences) panel administered by CentERdata (Tilburg University, Netherlands). The LISS panel consists of a representative sample of 5000 Dutch households that were randomly selected from municiipal registers in the Netherlands. We used data from a specific study module on mental health and flourishing (Lamers et al., 2011, 2015). In one third of the households, one member was selected by CentERdata to fill out this module (*n* = 1662, response rate 69%). To this dataset we linked data from a LISS core module on health that contained data on pain and relevant health-related variables, and sociodemographic variables (final *n* = 1498). All questionnaires were administered between November and December 2007. People were categorized for this study as having chronic pain when they ‘regularly suffered from back-, knee-, hip-pain or pain in any other joint’ (47.4%, *n* = 710), and compared to those without chronic pain (52.6% of total sample, *n* = 788). As we could not ensure that all included participants in this generic pain sample would adhere to important classifications for chronic pain (e.g., a minimal duration of 3 or 6 months), we also more narrowly pinpointed this generic chronic pain sample by extracting the people that both ‘regularly suffered from back-, knee-, hip-pain or pain in any other joint’ and had ‘a diagnosis of arthritis, including osteoarthritis, rheumatism, or osteoporosis’ (7.5% of total sample, *n* = 113).

#### Measures

##### Sociodemographic and health-related characteristics

Sociodemographic characteristics were age, gender, educational level and marital status. Health-related characteristics were the number of comorbid somatic conditions/health risks (e.g., diabetes, cancer, cardiovascular disease, COPD/asthma, high cholesterol, or blood pressure) and the number of comorbid recurrent physical symptoms (e.g., fatigue, sleep problems, stomach, or intestinal problems).

##### Positive mental health

Positive mental health was measured with the Mental Health Continuum-Short Form (MHC-SF) (Keyes et al., 2008; Lamers et al., 2011) which consists of 14 items (Keyes, 2002). Respondents rate the frequency of every feeling in the past month on a six-point Likert scale (never, once or twice a month, about once a week, two or three times a week, almost every day, every day). Example items of the three types of well-being are: “During the past month, how often did you feel: … interested in life? (emotional well-being, three items); …that you had experiences that challenged you to grow and become a better person? (psychological well-being, six items); … that you had something important to contribute to society?” (social well-being, five items). The MHC-SF showed high internal consistency in this sample (α = 0.90). Respondents can be categorized into two groups: flourishing or not flourishing. Respondents are categorized as flourishing when they score one of the three items on the emotional well-being subscale as ‘every day’ (6) or ‘almost every day’ (5), and rate at least 6 of the 11 items on the psychological and social well-being scale as ‘every day’ (6) or ‘almost every day’ (5) (Keyes, 2002). The Dutch version has good psychometric properties in both general and psychiatric samples (Lamers et al., 2011, 2012; Franken et al., 2018).

##### Psychopathological symptoms

The Brief Symptom Inventory (BSI; Dutch version) is a 53-item instrument for screening and assessment of psychopathology (De Beurs and Zitman, 2005). Respondents indicate the degree to which they experienced various psychological symptoms in the past week using a five-point Likert scale, ranging from 1 (not at all) to 5 (a lot). The BSI includes nine subscales: Depression, Anxiety, Phobic Anxiety, Interpersonal Sensitivity, Psychoticism, Paranoid Ideation, Hostility, Obsessive-compulsive Complaints and Somatization. The BSI showed high internal consistency in the present study (α = 0.94). Respondents can be categorized in two categories using a cutoff of 0.50: with or without being at risk for psychopathology.

#### Statistical Analyses

The Statistical Program for Social Sciences (IBM SPSS) version 24.0 was used for all statistical analyses. We used an alpha level of 0.05 for all statistical tests. Frequency distributions were calculated and descriptive analyses were performed to summarize sample characteristics of three groups beyond the total sample (*n* = 1498): People with chronic pain (*n* = 597), people with chronic pain in the context of arthritis (*n* = 113), and people without chronic pain (*n* = 788). Chi-square tests and independent *t*-tests were used to compare sample characteristics for the two subsamples with chronic pain with the subsample without chronic pain. Normality tests showed that three outliers were present for the variable ‘number of comorbid somatic conditions/health risks’ (reporting ≥ 12 comorbid somatic conditions). As findings were similar for data with these outliers removed and the full sample, only the outcomes for the full sample were reported. To examine the prevalence of *flourishing* for individuals within the two subsamples with chronic pain in comparison to individuals in the subsample without chronic pain, we first determined for each subsample the percentage of respondents in each category of positive mental health (i.e., flourishing, or not) and risk for psychopathology (i.e., at risk, or not). Hereafter, a chi-square test was performed to examine the prevalence of flourishing for individuals with chronic pain in comparison to people without chronic pain. Similarly, a chi-square test was performed to examine the prevalence of being *at risk for psychopathology* for individuals with chronic pain in comparison to people without chronic pain. All expected cell counts during chi-square tests were greater than five.

### Results

Overall, Chi-square tests and independent *t*-tests used to compare sample characteristics for the groups with and without chronic pain (Table 1) showed that both subsamples with chronic pain significantly differed from the group without chronic pain on all included sociodemographic and health-related characteristics (all *p*’s < 0.05). Compared to participants with*out* chronic pain, people with chronic pain were relatively older, more often female, received less education, were more often married, and suffered from more comorbid somatic conditions/health risks and comorbid recurrent physical symptoms. The same, but more skewed picture could be drawn for the participants with chronic pain in the context of arthritis.

**TABLE 1:** Descriptive characteristics of the total sample and subsamples with chronic pain, chronic pain in the context of arthritis, or without chronic pain (Study 1).

	**Chronic pain (*n* = 597) %**	**Arthritis (*n* = 113) %**	**No chronic pain (*n* = 788) %**	**Total sample (*n* = 1498) %**
**Sociodemographic**				
Age (mean, sd)	^∗^49.8 (17.1)	^∗^61.3 (12.0)	44.9 (17.8)	48.1 (17.7)
Gender				
Female	^∗^48.2	^∗^77.0	45.2	49.8
Education level				
Low	^∗^47.4	^∗^58.0	37.6	43.0
Intermediate	41.8	33.9	48.8	44.9
High	10.8	8.1	13.6	12.1
Marital status				
Married	^∗^57.8	^∗^62.8	48.9	53.5
**Health**				
Comorbid somatic conditions^a^				
0	^∗^49.1	^∗^26.5	67.9	57.3
1	33.2	33.6	23.5	28.1
2	10.9	21.2	6.9	9.5
3 or more	6.8	18.7	1.7	5.1
Comorbid recurrent physical symptoms^b^				
0	^∗^30.0	^∗^10.6	44.4	36.1
1	28.0	20.4	26.8	26.8
2	17.4	20.4	17.9	17.9
3	10.2	18.6	6.6	8.9
4 or more	14.4	30.0	4.3	10.3

*^∗^Significant difference in comparison with subsample without chronic pain (*p* < 0.05). ^*a*^Most prevalent comorbid somatic conditions/health risks in total sample were high blood pressure, high cholesterol, diabetes, and COPD/asthma. ^*b*^Most prevalent comorbid recurrent physical symptoms in the total sample were fatigue, flu-related complaints, headache, and sleeping problems.*

#### Prevalence of Flourishing and Being at Risk for Psychopathology in People With and Without Chronic Pain

**FIGURE 1:**
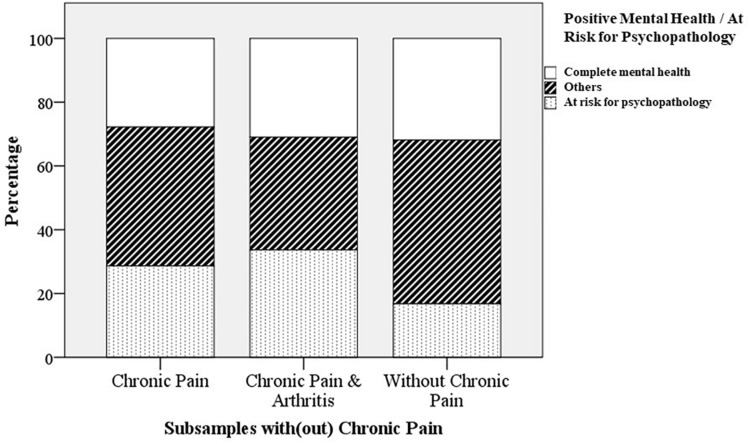
Prevalence of being at risk for psychopathology and complete mental health (Keyes, 2002); flourishing and not at risk for psychopathology) in different subsamples with or without chronic pain (Study 1).

The prevalence of flourishing in the total sample was 34.9% (Table 2). Whereas 34.0% of the participants with chronic pain and 38.1% of the participants with chronic pain and arthritis was flourishing, the prevalence of flourishing was 35.2% for the participants with*out* chronic pain. A chi-square test showed that the prevalence rates of flourishing did not differ significantly between the subsamples with and without chronic pain [χ^2^(2, *n* = 1498) = 0.728, *p* = 0.695, also the χ^2^-tests for a direct comparison of either of both subsamples with chronic pain versus the subsample without chronic pain separately, *p* > 0.05]. The mean prevalence of being at risk for psychopathology was 22.8% in the total sample, 28.6% for those with chronic pain, 33.6% for those with chronic pain and arthritis and 16.8% for the participants with*out* chronic pain. These prevalence rates differed significantly between the three subsamples [χ^2^(2, *n* = 1498) = 36.084, *p* < 0.001]. *Post hoc* tests with a direct comparison of the groups showed that both the subsample with chronic pain [χ^2^(1, *n* = 1358) = 29.364, *p* < 0.001] and the subsample with chronic pain and arthritis [χ^2^(1, *n* = 889) = 17.613, *p* < 0.001] were significantly more at risk for psychopathology than the subsample without chronic pain. Figure 1 displays the prevalence of *complete mental health* (i.e., not at risk for psychopathology and flourishing: Keyes, 2002) as well as the prevalence of being at risk for psychopathology for the different subsamples with and without chronic pain. The mean prevalence of complete mental health in the total sample and subsamples was slightly lower compared to the prevalence of flourishing (that could co-occur with being at risk for psychopathology) and ranged from 27.8% (subsample with chronic pain) to 31.9% (subsample without chronic pain). Also the prevalence of *complete mental health* did not significantly differ between the subsamples with and without chronic pain [χ^2^(2, *n* = 1498) = 2.678, *p* = 0.262, also the χ^2^-tests for a direct comparison of either of both subsamples with chronic pain versus the subsample without chronic pain separately, *p* > 0.05].^1^

**TABLE 2:** Prevalence of flourishing and being at risk for psychopathology in the total sample and subsamples with and without chronic pain (Study 1).

	**Chronic pain (*n* = 597) %**	**Arthritis (*n* = 113) %**	**No chronic pain (*n* = 788) %**	**Total sample (*n* = 1498) %**
**Mental health**				
Flourishing	34.0	38.0	35.2	34.9
Not flourishing	66.0	62.0	64.8	65.1
**At risk for psychopathology**				
At risk	28.6	33.6	16.8	22.8
Not at risk	71.4	66.4	83.2	77.2

## Study 2

### Materials and Methods

#### Participants and Procedure

This study draws on baseline data from a Randomized Controlled Trial on the effectiveness of web-based Acceptance and Commitment Therapy (ACT) for chronic pain (see Trompetter et al., 2015b, for a detailed description of study design and outcomes). The study protocol was approved by the Dutch Medical-Ethical Review Board (METC, trial number NL38622.044.11), which operates under the Dutch Central Committee for Research involving human participants (CCMO). The study has been registered in the Dutch Trial Register (Nederlands Trialregister), the primary trial register for clinical trials in Netherlands (trial number NTR3659). Participants were 238 people with chronic pain recruited in 2012 via national newspaper advertisements and online patient platforms from the Dutch general population. Diagnoses were heterogeneous and primarily included people without a chronic pain diagnosis, fibromyalgia, back pain, rheumatic diseases, and neuropathic pain. Study inclusion criteria were being 18 years or older, having a momentary pain intensity Numeric Rating Scale (11-point NRS) score > 4, and having pain for at least 3 days per week for at least 6 months. Exclusion criteria were severe psychological distress as assessed with the Hospital Anxiety and Depression Scale (HADS; score > 24) (Zigmond and Snaith, 1983), extremely low levels of psychological inflexibility as assessed with the Psychological Inflexibility in Pain scale (PIPS; score < 24) (Wicksell et al., 2010a), participation in another Cognitive Behavioral Treatment, having no internet or e-mail address, reading problems in Dutch language, and/or an unwillingness or inability to invest time. Participants filled in the baseline data via an online questionnaire, either during (e.g., HADS) or after checks (e.g., MHC-SF) of in- and exclusion criteria and always prior to randomization procedures.

#### Measures

##### Sociodemographic variables

Sociodemographic variables were *age*, *gender*, *educational level* (low, medium, high) and *marital status* (married/living together or not).

##### Physical variables

Physical variables were pain intensity, pain duration and pain disability. *Pain intensity* was measured with a 11-point Numeric Rating Scale (NRS), ranging from ‘no pain’ (0) to ‘pain as bad as you can imagine’ (10) (Dworkin et al., 2005). *Pain duration* was categorized into a dummy variable (less/more than 5 years). *Pain disability* was measured with the Pain Disability Index (PDI) (Pollard, 1984), that consists of seven items and assesses the degree to which chronic pain disables a person from performing daily activities such as work (total score range 7–70) (α = 0.81).

##### Positive mental health

Positive mental health and flourishing were measured and categorized in the same way as in Study 1. Internal consistency of the MHC-SF was high in the current study (α = 0.89).

##### Depressive symptoms

Depressive symptoms were measured with the depressive symptom subscale of the HADS (Zigmond and Snaith, 1983). The scale consists of seven items, and measures the presence and severity of depressive symptoms (total score range 0–21) (α = 0.79). Individuals with a HADS score ≥ 8 are at risk for depression.

##### (Other) Psychological variables

Psychological variables were pain catastrophizing, psychological inflexibility, mindfulness and engaged living. *Pain catastrophizing* was measured with the Pain Catastrophizing Scale (PCS), a 13-item questionnaire (Sullivan et al., 1995). The scale measures levels of pain rumination, magnification, and helplessness (total score range 0–52) (α = 0.91). *Psychological inflexibility* was measured with the PIPS that consists of 12 items (Wicksell et al., 2010a). The scale measures experiential avoidance of pain and cognitive fusion with pain-related thoughts and feelings (total scale range 12–84) (α = 0.87). *Mindfulness* was measured with the Five Facet Mindfulness Questionnaire—Short Form (FFMQ-SF). The FFMQ-SF (Baer et al., 2006; Bohlmeijer et al., 2011) is a 24-item questionnaire that measures five facets of mindfulness: observing, describing, acting with awareness, non-judging and non-reactivity (total score range 24–120) (α = 0.82). Finally, *engaged living* was measured with the Engaged Living Scale (ELS) (Trompetter et al., 2013) that consists of 16 items. The ELS measures the extent to which people know and act upon their personal values, and experience a sense of fulfillment in life as a consequence of doing so (total scale range 16–80) (α = 0.91).

#### Statistical Analyses

The Statistical Program for Social Sciences (IBM SPSS) version 24.0 was used for all statistical analyses. We used an alpha level of 0.05 for all statistical tests. Frequency distributions were calculated and descriptive analyses were performed to summarize sample characteristics of the total chronic pain sample (*n* = 238). First, the prevalence of flourishing was determined as well as the prevalence of being at risk for depression. Chi-square tests and independent *t*-tests were used to compare functioning on all individual sociodemographic, physical and psychological correlates for flourishers (1) versus non-flourishers (0), and for being at risk for depression (1) versus not being at risk for depression (0). Then, we performed a multivariate stepwise logistic regression analysis with (not) flourishing (MHC-SF) as the dependent variable. Potential correlates were entered in blocks. After controlling for the level of depressive symptoms (HADS), sociodemographic (1), physical (2) and psychological variables (3) were entered. This hierarchical inclusion enabled us to assess the individual contribution to identify flourishers for each variable set. This multivariate stepwise logistic regression analysis was repeated with (not) being at risk for depression as the dependent variable. In a first step, we controlled for levels of positive mental health in this analysis. In both models we only included the correlates on which flourishers and non-flourishers, and/or those either at risk and not at risk for depression differed (marginally) significantly in previous chi-square tests and independent *t*-tests. All continuous correlates were normally distributed and did not include any outliers. Also inspection of residuals following the logistic regression analysis suggested no influential outliers in the data. Finally, outcomes of Pearson’s correlation coefficients also suggested that none of the included correlates showed multicollinearity with each other (all *r* < 0.57). We reported beta’s, standard errors and odds ratio’s [exp (B)] for each correlate, as well as Nagelkerke R^2^ as a measure of the effect size for each block of variables and the final, full model. The reported coefficients for individual correlates can be interpreted as such that – holding the other correlates constant at their mean values (continuous) or lowest values (dichotomous) – a change of one unit in this correlate will change the odds of *y* (i.e., flourishing) by a factor of exp (B). We transpose this one-unit change to a change of 1 SD for significant, continuous correlates to improve interpretation of the results.

### Results

#### Prevalence of Flourishing and Being at Risk for Depression

The typical study participant was a middle aged, higher educated female in a relationship, whom suffered on a daily basis from pain [mean pain intensity levels: 6.2 (*SD* = 1.7)] for over 5 years (Table 3). The prevalence of flourishing was 22.8%, while the prevalence of being at risk for depression was 35.7%.

**TABLE 3:** Descriptive characteristics of the sample (Study 2).

	**Total sample (*n* = 238) %/M (*SD*)**
Flourishing (%)	22.8
At risk for depression (%)	35.7
**Demographic characteristics**	
Age	52.8 (12.4)
Female (%)	76.1
**Education level (%)**	
Low	20.2
Intermediate	35.7
High	44.1
Married/living together (%)	74.4
**Physical characteristics**	
Pain duration > 5 years (%)	63.0
Pain intensity (11-point NRS)	6.2 (1.7)
Pain disability (PDI)	36.2 (12.6)
**Psychological characteristics**	
Pain catastrophizing (PCS)	18.4 (19.8)
Psychological inflexibility (PIPS)	54.9 (11.5)
Mindfulness (FFMQ)	81.7 (10.7)
Engaged living (ELS)	50.9 (9.8)

#### Correlates of Flourishing and Being at Risk for Depression

Chi-square tests and independent *t*-tests comparing flourishers and non-flourishers with each other on each individual sociodemographic, physical and psychological correlate (Table 4) revealed that both groups did not significantly differ on any of the sociodemographic variables (all *p*’s > 0.10). With regard to the physical correlates, flourishers (*M* = 32.4, *SD* = 13.0) scored significantly lower than non-flourishers on pain disability; *t*(236) = 2.478, *p* = 0.014. Both groups also scored significantly different on all psychological correlates. Flourishers were less psychologically inflexible (*M* = 50.4, *SD* = 11.1) than non-flourishers (*M* = 56.1, *SD* = 12.0); *t*(236) = 3.230, *p* = 0.001, and experienced lower levels of pain catastrophizing (flourishers, *M* = 16.1, *SD* = 10.0; non-flourishers, *M* = 19.1, *SD* = 9.6); *t*(236) = 1.966, *p* = 0.050. In contrast, flourishers (*M* = 86.9, *SD* = 10.4) experienced higher levels of mindfulness than non-flourishers (*M* = 80.2, *SD* = 10.4); *t*(236) = −4.173, *p* < 0.001, and engaged living (flourishers, *M* = 58.8, *SD* = 8.7; non-flourishers, *M* = 48.6, *SD* = 8.9); *t*(236) = −7.391, *p* < 0.001. People (not) at risk for depression differed significantly on the same correlates, but with different patterns. With regard to the physical correlates, those at risk for depression scored significantly higher (*M* = 39.9, *SD* = 10.6) than those not at risk for depression (*M* = 34.1, *SD* = 13.2) on pain disability; *t*(236) = −3.490, *p* < 0.001. People at risk for depression were more psychologically inflexible (*M* = 61.1, *SD* = 10.6) than people not at risk for depression (*M* = 51.4, *SD* = 10.6); *t*(236) = −6.818, *p* < 0.001, and experienced higher levels of pain catastrophizing (at risk, *M* = 23.3, *SD* = 9.2; not at risk, *M* = 15.7, *SD* = 9.0); *t*(236) = −6.146, *p* < 0.001. In addition, those at risk (*M* = 77.9, *SD* = 9.2) experienced lower levels than those not at risk (*M* = 83.8, *SD* = 11.0) of mindfulness; *t*(236) = 4.184, *p* < 0.001, and engaged living (at risk, *M* = 46.5, *SD* = 7.5; not at risk, *M* = 53.4, *SD* = 10.1); *t*(236) = 5.527, *p* < 0.001. Both groups did not score differently on any of the sociodemographic variables. *Marginally* significant differences existed, however, for the distribution of men and women over both groups, with more men than women present in the group at risk for depression than the group not at risk for depression; χ^2^(1, *n* = 238) = 3.199, *p* = 0.074.

**TABLE 4:** Descriptive statistics for sociodemographic, physical and psychological variables, and comparison on these variables for flourishers and non-flourishers, and those either at risk or not at risk for depression, respectively (Study 2).

	**Positive mental health**		**Depressive symptoms**	
	**Flourishing (*n* = 53) Mean (*SD*)**	**Not flourishing (*n* = 185) Mean (*SD*)**	**At risk (*n* = 85) Mean (*SD*)**	**Not at risk (*n* = 153) Mean (*SD*)**
**Sociodemographic variables**				
Age	52.6 (11.3)	52.8 (12.7)	54.4 (12.0)	51.6 (12.5)
Female (%)^a^	77.4	75.7	69.4^X^	79.7
High education level (%)^b^	41.5	44.9	41.2	45.8
Married/living together (%)^c^	83.0	71.9	80.0	71.2
**Physical variables**				
>5 years pain duration (%)^d^	71.7	60.5	65.9	61.4
Pain intensity (11-point NRS)	6.5 (1.6)	6.1 (1.7)	6.5 (1.5)	6.0 (1.7)
Pain disability (PDI)	32.4 (13.0)^∗^	37.2 (12.4)	39.9 (10.6)^∗∗^	34.1 (15.7)
**Psychological variables**				
Pain catastrophizing (PCS)	16.1 (10.0)^∗^	19.1 (9.6)	23.3 (9.2)^∗∗^	15.7 (9.0)
Psychological inflexibility (PIPS)	50.4 (12.0)^∗∗^	56.1 (11.1)	61.1 (10.6)^∗∗^	51.4 (10.6)
Mindfulness (FFMQ)	86.9 (10.4)^∗∗^	80.2 (10.4)	77.9 (9.2)^∗∗^	83.8 (11.0)
Engaged living (ELS)	58.8 (8.7)^∗∗^	48.6 (8.9)	46.5 (7.5)^∗∗^	53.4 (10.1)

*Chi-square and independent *t*-tests were performed to compare flourishers with non-flourishers (1), and to compare being at risk for depression with not being at risk for depression (2). ^∗^Significant at level *p* < 0.05; ^∗∗^Significant at level *p* < 0.01. ^*X*^Marginally significant at level *p* < 0.10. ^*a*^Dummy coded, 1 = female; ^*b*^dummy coded, 1 = high education level; ^*c*^dummy coded, 1 = married/living together; ^*d*^dummy coded, 1 ≥ 5 years pain duration.*

Results of the multivariate stepwise logistic regression analysis showed that the full, final model with socio-demographic, physical and psychological correlates significantly explained 30% of the variance in flourishing (Omnibus χ^2^ = 51.105, df = 7, *p* < 0.001). Levels of depressive symptoms (step 1) explained 14% of the variance in flourishing (Table 5). We found that *only* the psychological correlates (step 4) were related to flourishing in the final model with an additional R^2^ of 15%. Of the individual correlates, only *engaged living* (*B* = 0.09, OR = 1.10, 95% CI: 1.05–1.15) was significantly associated with flourishing, with higher levels of engaged living increasing the likelihood to flourish. The odds to flourish increase by a factor of 2.48 for each standard deviation increase in engaged living. The full, final model with socio-demographic, physical and psychological correlates significantly explained 37% of the variance in being at risk for depression (Omnibus χ^2^ = 74.989, df = 7, *p* < 0.001). Levels of positive mental health (step 1) explained 14% of the variance in being at risk for depression (Table 5). We found that *only* the psychological correlates (step 4) were related to flourishing in the final model with an additional R^2^ of 18%. Of the individual correlates, *pain catastrophizing* (*B* = 0.04, OR = 1.05, 95% CI: 1.01 – 1.09), *psychological inflexibility* (*B* = 0.05, OR = 1.05, 95% CI: 1.02 – 1.09) and *engaged living* (*B* = −0.05, OR = 0.94, 95% CI: 0.91–0.99) were significantly associated with being at risk for depression. Whereas *higher* levels of pain catastrophizing and psychological inflexibility were associated with a higher likelihood to be at risk for depression, *lower* levels of engaged living increasing the likelihood to be at risk for depression. The positive coefficients for pain catastrophizing and psychological inflexibility correspond to an increase in odds of being at risk for depression of 1.54 and 1.81 for each standard deviation increase in both correlates, respectively. The size of the coefficient for engaged living means that one standard deviation increase in engaged living decreases the odds to be at risk for depression with a factor 0.60. An overall integration of the outcomes of logistic regression analyses and the descriptive patterns on the included correlates suggests that engaged living particularly sets apart flourishers from the other mental health groups, while pain catastrophizing and psychological inflexibility particularly set apart those at risk for depression.

**TABLE 5:** Logistic regression coefficients associated with flourishing and being at risk for depression (Study 2).

		**Flourishing**		**Being at risk for depression**	
		**B (S.E.)**	**OR (95% CI)**	**B (S.E.)**	**OR (95% CI)**
*Step 1*	Depressive symptoms (HADS)/positive mental health (MHC-SF)	−0.09(0.08)	0.92 (0.79; 1.06)	−0.19(0.24)	0.82 (0.52; 1.31)
			*R*^2^ = 0.14		*R*^2^ = 0.14
*Step 2*	Gender^a^	−0.04(0.44)	0.96 (0.40; 2.30)	0.64 (0.39)	1.90 (0.89; 4.09)
			*R*^2^ = 0.14		*R*^2^ = 0.14
*Step 3*	Pain disability (PDI)	−0.01(0.02)	0.99 (0.96; 1.02)	0.02 (0.02)	1.02 (0.99; 1.05)
			*R*^2^ = 0.15		*R*^2^ = 0.19
*Step 4*	Pain catastrophizing (PCS)	0.01 (0.02)	1.01 (0.97; 1.06)	0.04^∗^(0.02)	1.05 (1.01; 1.09)
	Psychological inflexibility (PIPS)	−0.01(0.02)	0.96 (0.96; 1.04)	0.05^∗^(0.02)	1.05 (1.02; 1.09)
	Mindfulness (FFMQ)	0.03 (0.02)	1.03 (0.99; 1.07)	−0.03(0.02)	0.97 (0.94; 1.02)
	Engaged living (ELS)	0.09^∗^(0.02)	1.10 (1.05; 1.15)	−0.05^∗^(0.02)	0.94 (0.91;0.99)
			*R*^2^ = 0.30		*R*^2^ = 0.37

*Results are for the final model (step 4). Explained variance is Nagelkerke R^2^. ^∗^Significant at level *p* < 0.05. ^*a*^Dummy coded, 1 = female.*

## Discussion

This study aimed to explore the prevalence and correlates of positive mental health for people with chronic pain and (being at risk for) psychopathology, and in comparison to people with*out* chronic pain. The prevalence of flourishing was 34% in the general population sample with recurrent joint pain, 38% in the general population sample with arthritis, and 23% in our chronic pain sample seeking help. People with chronic pain in the general population either with or without arthritis were as likely to flourish as people with*out* chronic pain. In contrast, the prevalence of being at risk for psychopathology for people with chronic pain was elevated compared to people without chronic pain. Finally, both flourishing and being at risk for depression were related foremost to psychological correlates. While engaged living was the most important correlate of flourishing, pain catastrophizing, psychological inflexibility, and engaged living were the most important correlates of being at risk for depression. Correlates of both mental health outcomes are thus partly overlapping, but simultaneously unique correlates emerged and all correlates had differential patterns of associations with both mental health outcomes. These findings support that positive and negative health- and well-being outcomes are related, but different constructs that function on two different continua (Huppert and Whittington, 2003; Schleicher et al., 2005; Huber et al., 2008; Westerhof and Keyes, 2009). Our findings on the prevalence and elevated chances of being at risk for psychopathology are in line with our hypotheses and existing research (e.g., Miller and Cano, 2009; de Heer et al., 2018). de Heer et al. (2018) reported that moderate to very severe pain was associated with a twofold risk of mood and anxiety disorders in the Dutch general population. Similarly existing research suggested that – although a significant subgroup of people with chronic pain in the general population *is able* to flourish – on average people with chronic pain are less likely flourish than people without chronic pain (Keyes, 2005a; Gilmour, 2015). We thus revealed different findings. As the sample of Gilmour (2015) resembled our broad, generic population sample, Keyes (2005a) actually included a specific chronic pain sample very similar to our subsample from the general population with arthritis. It is thus unlikely that we can attribute the differential findings between our and their studies to differences chronic pain sampling procedures. As proposed by Schotanus-Dijkstra et al. (2016), cultural factors like socio-economic advantages, and higher levels of individualism and social equality positively influence national levels of emotional well-being (Diener et al., 1995). These factors might aid people in the Dutch general population in contrast to other cultures to experience high positive mental health in the presence of chronic pain and arthritis. Individual variation in levels of positive mental health and being at risk for depression was explained primarily by psychological factors. Research and practice has long recognized the pivotal role of psychological factors in pain-related health and well-being (McCracken and Turk, 2002; Gatchel et al., 2007; Jensen and Turk, 2014). We know, for example, that pain acceptance is more important in explaining pain-related health and well-being outcomes than pain intensity levels (Viane et al., 2003; McCracken and Eccleston, 2005; Vowles et al., 2007; McCracken and Gutiérrez-Martínez, 2011). Also, behavioral interventions for chronic pain are only effective when they additionally targets psychological factors (Williams et al., 2013). Our study reveals several additional findings. Higher levels of engaged living was the most important correlate of flourishing. These findings resonate with the inherent focus of the psychological flexibility model (PF) on positive mental health. Particularly, its unique, person-oriented focus on long-term engagement with personally valued activities in the presence of persistent pain (McCracken and Vowles, 2014; Goubert and Trompetter, 2017). On the other hand, the most important correlates of being at risk for depression were pain catastrophizing, psychological inflexibility and engaged living. An overall integration of the outcomes suggests that particularly pain catastrophizing and psychological inflexibility set apart those at risk for depression. Both factors are related indeed to each other and to negative outcomes in and outside the context of chronic pain (Vowles et al., 2007; Aldao et al., 2010; Wicksell et al., 2010b; Trompetter et al., 2015a). An overall integration of findings supports that core therapeutic processes of the PF model – psychological inflexibility and engaged living – in combination provide pathways to decrease both depressive symptoms and simultaneously enhance positive mental health in chronic pain patients (McCracken and Morley, 2014; Goubert and Trompetter, 2017). Simultaneously, the FA model seems to particularly explain being at risk for depression and not flourishing. ACT interventions based on the PF model have been able to change both mental health outcomes successfully in people with mild depressive symptoms (Fledderus et al., 2012). While some people improved on both mental health outcomes, some improved on either one of both (Trompetter et al., 2017). On the contrary, the same ACT intervention reworked for people with chronic pain was able to enhance depressive symptoms but not positive mental health (Trompetter et al., 2015b). Knowledge is fully lacking at present to explain these differential effects within and between diagnostic groups. Furthermore, positive and negative correlates and outcomes of health and well-being in the context of chronic pain are related, but different constructs that function on two different continua. It is likely that other, unique protective psychological factors exist that function as important correlates of flourishing in the context of chronic pain. For example, positive affect and optimism (Zautra et al., 2001; Finan et al., 2013; Hanssen et al., 2014; Finan and Garland, 2015). Finally, a lower percentage of people with chronic pain flourished in our second sample (23%). This is plausible as the sample was explicitly targeted for their need to help reduce the interference of chronic pain with daily life activities. Our findings do suggest that people with chronic pain are open to receive or seek help – at least via an easily accessible Internet-based intervention (Trompetter et al., 2015b) – because they experience low levels of well-being beyond high levels of negative pain-related outcomes. Improvements in well-being have proven to be at least as important as relief from symptoms of psychopathology for patient groups with major depression (Zimmerman et al., 2006; Demyttenaere et al., 2015). It is interesting to further explore chronic pain patients viewpoints on desired treatment outcomes in the future. This study has several limitations. It concerns a *post hoc*, exploratory study. This limited Study 1 primarily in our operationalization of chronic pain. We do not know if our sample with chronic (joint) pain adhered to important classifications for chronic pain like a minimal duration of 3 or 6 months. Both pain duration and intensity were, however, not associated with flourishing in Study 2, and all findings in Study 1 were similar for the sample with rheumatoid arthritis as well as the more generic pain sample. A limitation of Study 2 is that we could not include several protective psychological factors that may function as important correlates of (positive) mental health in the context of chronic pain (e.g., positive affect, optimism). A further limitation is the fact that both studies were cross-sectional. Thus, we cannot draw conclusions regarding causality between levels of positive mental health, depressive symptoms, chronic pain and included psychological correlates. Finally, both included chronic pain samples were very heterogeneous in nature. We do not know if and to what extent our findings generalize to specific diagnostic groups with chronic pain or people with clinical levels of depression. Previous studies for example showed that fibromyalgia patients, but not patients with rheumatoid arthritis, function worse on aspects of positive mental health compared to healthy subgroups (Schleicher et al., 2005; Finan et al., 2009). This study suggests that negative and positive mental health outcomes and their correlates in the context of chronic pain are related but simultaneously of a different, unique nature. Also, a relatively large group of people with chronic pain is able to maintain high levels of positive mental health. We hope that our outcomes boost further research on protective beyond risk pain-related factors and positive beyond negative pain-related outcomes, particularly the study of unique, protective mechanisms involved in the ability to maintain or achieve optimal levels of positive mental health in the presence of chronic pain and other disabilities.

## Data Availability Statement

The data for Study 1 uses LISS (Longitudinal Internet Studies for the Social sciences) panel data, administered by CentERdata (Tilburg University, Netherlands). LISS panel data is freely available for scientific purposes under specific conditions (www.lissdata.nl). Information on variables used from LISS and the dataset generated for Study 2 are available on request to the corresponding author.

## Ethics Statement

All patients/participants provided their written informed consent. The original study that provided data for Study 2 was reviewed and approved by the Dutch Medical-Ethical Review Board (METC, trial number NL3622.044.11).

## Author Contributions

HT executed the original study that provided data for in Study 2, designed the study, performed data analysis and interpretation of the data, and drafted the manuscript. FM was involved in decisions on data analysis, data interpretation, and manuscript writing. GW was responsible for conception of the original study that provided data for Study 1, and contributed to decisions on data analysis, data interpretation, and manuscript writing. All authors contributed to critical revisions of the manuscript and approved the final version of the manuscript.

## Conflict of Interest

The authors declare that the research was conducted in the absence of any commercial or financial relationships that could be construed as a potential conflict of interest.

## Footnotes

1 Both chronic pain samples differed from the sample without chronic pain on each of the sociodemographic and health-related assessed. We thus performed *post hoc* multivariate logistic regression analyses to examine the association between chronic pain and both mental health outcomes while controlling for the included sociodemographic and health-related variables. The results were similar to the reported findings. The subsample with chronic pain, as well as the subsample with chronic pain and arthritis, were as likely to flourish as the sample without chronic pain [*B* = 0.09, S.E. (B) = 0.12, *p* = 0.42 and *B* = 0.18, S.E. (B) = 0.12, *p* = 0.13 respectively]. Simultaneously, the subsample with chronic pain as well the subsample with chronic pain and arthritis were more likely to be at risk for psychopathology than the sample without chronic pain [*B* = 0.71, S.E. (B) = 0.14, *p* < 0.01 and *B* = 0.44, S.E. (B) = 0.14, *p* < 0.01 respectively].
